# A combinational therapy of EGFR-CAR NK cells and oncolytic herpes simplex virus 1 for breast cancer brain metastases

**DOI:** 10.18632/oncotarget.8526

**Published:** 2016-04-01

**Authors:** Xilin Chen, Jianfeng Han, Jianhong Chu, Lingling Zhang, Jianying Zhang, Charlie Chen, Luxi Chen, Youwei Wang, Hongwei Wang, Long Yi, J. Bradley Elder, Qi-En Wang, Xiaoming He, Balveen Kaur, E. Antonio Chiocca, Jianhua Yu

**Affiliations:** ^1^ Division of Hematology, Department of Internal Medicine, College of Medicine, The Ohio State University, Columbus, Ohio 43210, USA; ^2^ Lymphoma/Head and Neck Oncology Department, 307 Hospital, Beijing 100071, China; ^3^ The Ohio State University Comprehensive Cancer Center, Columbus, Ohio 43210, USA; ^4^ Institute of Blood and Marrow Transplantation, Soochow University, Suzhou 215000, China; ^5^ Center for Biostatistics, The Ohio State University, Columbus, Ohio 43210, USA; ^6^ Department of Radiology, The Ohio State University, Columbus, Ohio 43210, USA; ^7^ Department of Biomedical Engineering, The Ohio State University, Columbus, Ohio 43210, USA; ^8^ Department of Neurological Surgery, The Ohio State University, Columbus, Ohio 43210, USA; ^9^ Department of Neurosurgery, Brigham and Women's Hospital and Harvey Cushing Neuro-oncology Laboratories, Harvard Medical School, Boston, Massachusetts 02115, USA; ^10^ The James Cancer Hospital, Columbus, OH 43210, USA

**Keywords:** breast cancer brain metastases, chimeric antigen receptor, natural killer cells, EGFR, oncolytic virus

## Abstract

Breast cancer brain metastases (BCBMs) are common in patients with metastatic breast cancer and indicate a poor prognosis. These tumors are especially resistant to currently available treatments due to multiple factors. However, the combination of chimeric antigen receptor (CAR)-modified immune cells and oncolytic herpes simplex virus (oHSV) has not yet been explored in this context. In this study, NK-92 cells and primary NK cells were engineered to express the second generation of EGFR-CAR. The efficacies of anti-BCBMs of EGFR-CAR NK cells, oHSV-1, and their combination were tested *in vitro* and in a breast cancer intracranial mouse model. *In vitro*, compared with mock-transduced NK-92 cells or primary NK cells, EGFR-CAR-engineered NK-92 cells and primary NK cells displayed enhanced cytotoxicity and IFN-γ production when co-cultured with breast cancer cell lines MDA-MB-231, MDA-MB-468, and MCF-7. oHSV-1 alone was also capable of lysing and destroying these cells. However, a higher cytolytic effect of EGFR-CAR NK-92 cells was observed when combined with oHSV-1 compared to the monotherapies. In the mice intracranially pre-inoculated with EGFR-expressing MDA-MB-231 cells, intratumoral administration of either EGFR-CAR-transduced NK-92 cells or oHSV-1 mitigated tumor growth. Notably, the combination of EGFR-CAR NK-92 cells with oHSV-1 resulted in more efficient killing of MDA-MB-231 tumor cells and significantly longer survival of tumor-bearing mice when compared to monotherapies. These results demonstrate that regional administration of EGFR-CAR NK-92 cells combined with oHSV-1 therapy is a potentially promising strategy to treat BCBMs.

## INTRODUCTION

Breast cancer is the most common malignancy among females in the U.S. [1]. Metastasis is the major cause of mortality in breast cancer patients, with a total incidence of brain metastasis of about 30% [2]. Unfortunately, there is still no cure or safe treatments for such patients. The overall prognosis of patients with breast cancer brain metastases (BCBMs) remains very poor, with a median overall survival (OS) of 8.7 months and only 4.9 months for the triple-negative (ER–, PR–, HER2–) type [3]. Despite surgery, whole-brain radiation therapy, gamma-knife radiosurgery, and traditional chemotherapy, the poor survival underscores the urgent need for innovative and targeted gene therapies for BCBM patients.

Immunotherapy is a promising approach to control cancer progression, prolong patient's survival, and improve the quality of life, because immune effectors not only recognize and destroy tumor cells but also provide long-term immune surveillance. To date, a variety of immunotherapies, including cellular therapies, have been incorporated into cancer treatment. One of the most promising approaches is adoptive transfer of chimeric antigen receptor (CAR)-engineered T cells, in which patient T cells are engineered to specifically recognize a tumor antigen [4]. CAR NK cells have been designed to treat cancer since they may have a lower risk of inducing cytokine release syndrome, tumor lysis syndrome, as well as graft-versus-host disease (GVHD) in the allogeneic settings in patients [5], since CAR NK cells lack a clonal expansion and may have a different cytokine profile compared to CAR T cells. The main challenge for successful use of CAR NK cells and CAR T cells is to find a proper surface antigen to target. EGFRs are highly expressed in a majority of BCBM patients [6]. In addition to being present on some breast cancer stem cells (CSCs), EGFR plays an important role in cell proliferation, motility, and survival in various tumors including breast cancer [7]. Thus, it appears that EGFR may be a potential tumor antigen for CAR NK cells to target for the treatment of BCBMs.

As the first genetically engineered oncolytic virus (OV), oHSV-1 is an attractive vector for cancer gene therapy. oHSV-1 has a number of advantages over other OVs-derived viruses. For example, its genomic structure is very stable, and adverse events in patients can be counteracted with effective antiviral drugs [8, 9]. Moreover, various forms of oHSVs have already been applied in clinical trials against a wide range of cancers [10]. The U.S. FDA approved an engineered oHSV for the treatment of melanoma in October 2015. However, oHSVs are not very effective as a single agent to treat cancer so far. We have recently shown that oHSV therapy activated a host NK cell response against infected cells, which might in turn limit viral replication [11, 12].

Meanwhile, advances in cancer immunotherapy have inspired novel therapeutic strategies. Optimizing the effectiveness of immunotherapy should augment antitumor responses, and this may be achieved through combinational strategies. Theoretically, the combination of EGFR-CAR NK cell therapy and oHSV treatment may bear some advantages. Firstly, EGFR-expressing cancer cells will be efficiently targeted and lysed by EGFR-CAR NK cells, while oHSV-1 still has a chance to eradicate the remaining EGFR-negative or EGFR-dim cancer cells that may exist or are derived from EGFR-positive cells due to tumor antigen loss [13]. Secondly, CAR NK cells may destroy or loosen the tumor structure and therefore create an environment favorable for oHSV distribution and replication in cancer cells. Therefore, we hypothesized that CAR NK cell infusion followed by oHSV administration can improve the treatment of BCBMs.

Here, we generated CAR NK cells armed with an anti-EGFR single-chain variable fragment (scFv) with high antigen specificity and affinity, which is able to target both wild-type (wt) EGFR and EGFRvIII. We then investigated the potential of this targeted therapy, oHSV, and their combination for the treatment of EGFR-positive BCBMs.

## RESULTS

### Expression of EGFR in breast cancer cell lines and primary and metastatic tissues

**Figure 1:**
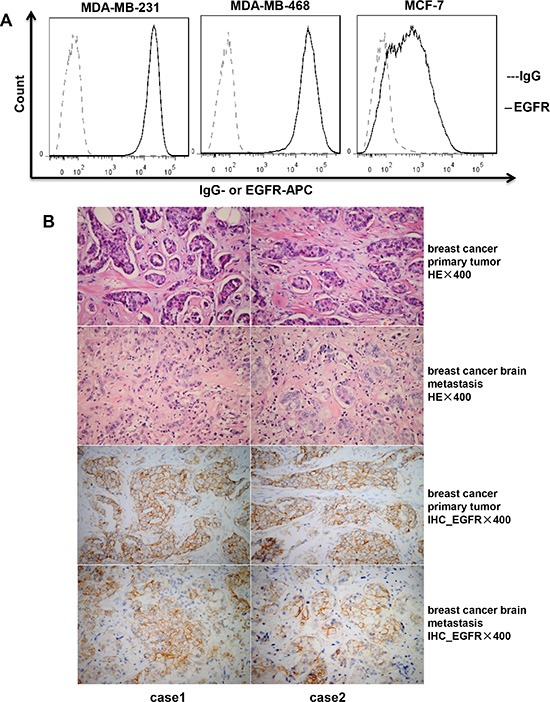
Expression of EGFR in breast cancer cell lines and tissues (**A**) Expression of EGFR on the cell surface of breast cancer cell lines (MDA-MB-231, MDA-MB-468, and MCF-7) detected by flow cytometry. (**B**) Hematoxylin and eosin (HE) staining and immunohistochemistry (IHC) of EGFR expression for tumor tissues from patients with primary breast cancer and brain metastases.

To assess the surface expression of EGFR in breast cancer cell lines, cells were stained with an EGFR-specific antibody, followed by flow cytometric analysis. As shown in Figure 1A, EGFR was expressed on the surface of MDA-MB-231, MDA-MB-468, and MCF-7 cell lines, although levels were clearly lower on MCF-7 cells. EGFR expression was then evaluated by immunohistochemistry (IHC) in primary tumor tissues and the corresponding brain metastasis lesions from two cases of patients diagnosed with metastatic breast cancer, after confirming the existence of tumor cells by hematoxylin and eosin (HE) staining (Figure 1B, top two rows). Surface EGFR expression was observed not only on tumor cells from the primary lesions, but also on those from the brain metastases (Figure 1B, bottom two rows).

### Enhanced cytotoxicity and IFN-γ production of EGFR-CAR NK-92 and primary NK cells

**Figure 2:**
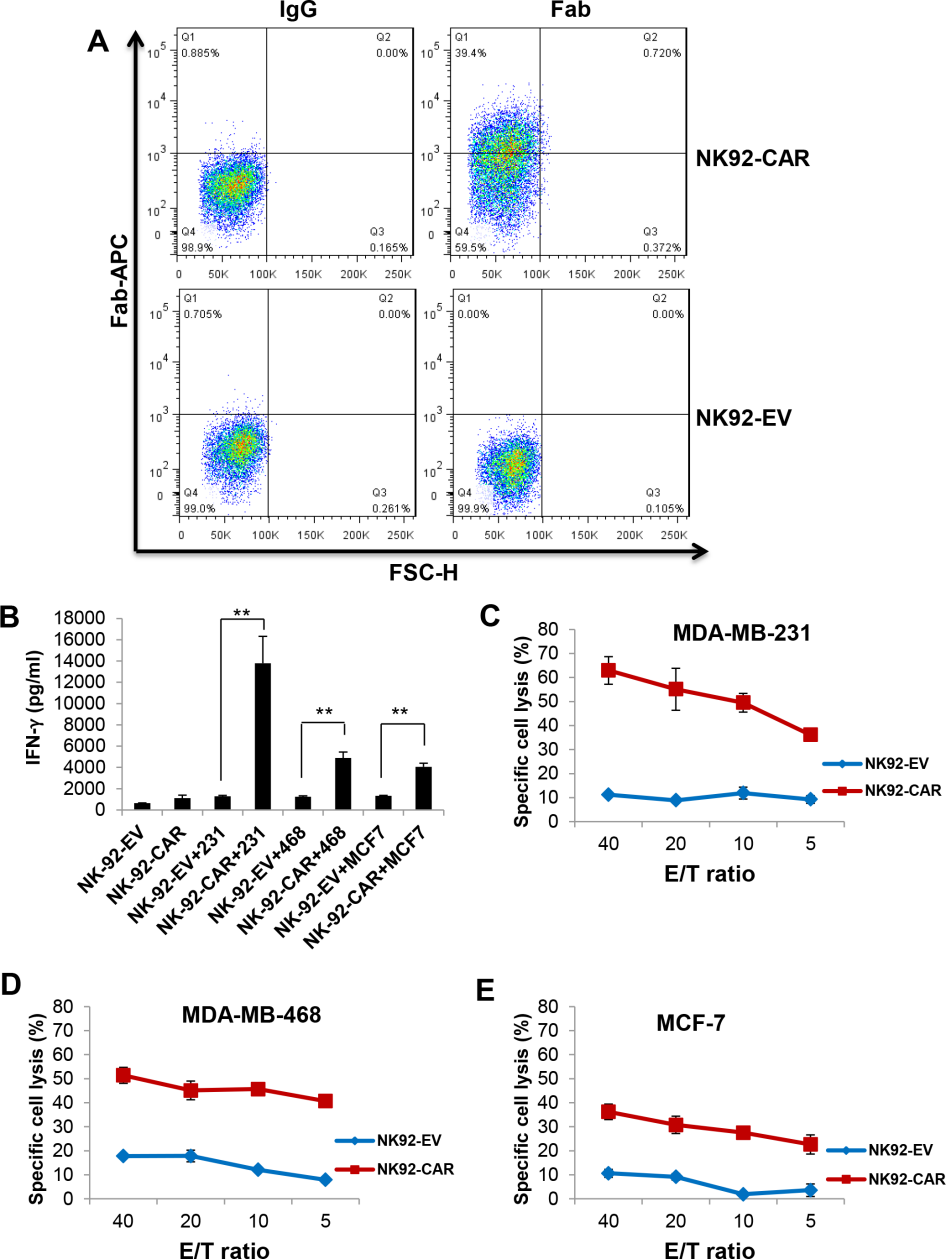
EGFR-CAR NK-92 cells recognize and lyse EGFR positive cells of breast cancer cell lines (**A**) Expression of EGFR scFv on EGFR-CAR-transduced NK-92 cells, determined by flow cytometry using a goat anti-mouse F(ab′)_2_ polyclonal antibody. (**B**) IFN-γ release by empty vector (EV)-transduced or EGFR-CAR-transduced NK-92 cells in the absence or presence of MDA-MB-231, MDA-MB-468 or MCF-7 cells using a standard ELISA assay. **P < 0.01. (**C**–**E**) Cytotoxic activity of empty vector (EV)-transduced or EGFR-CAR-transduced NK-92 cells against MDA-MB-231 (C), MDA-MB-468 (D), or MCF-7 (E) cells using a standard chromium-51 release assay. (E, effect cell; T, target cell).

**Figure 3:**
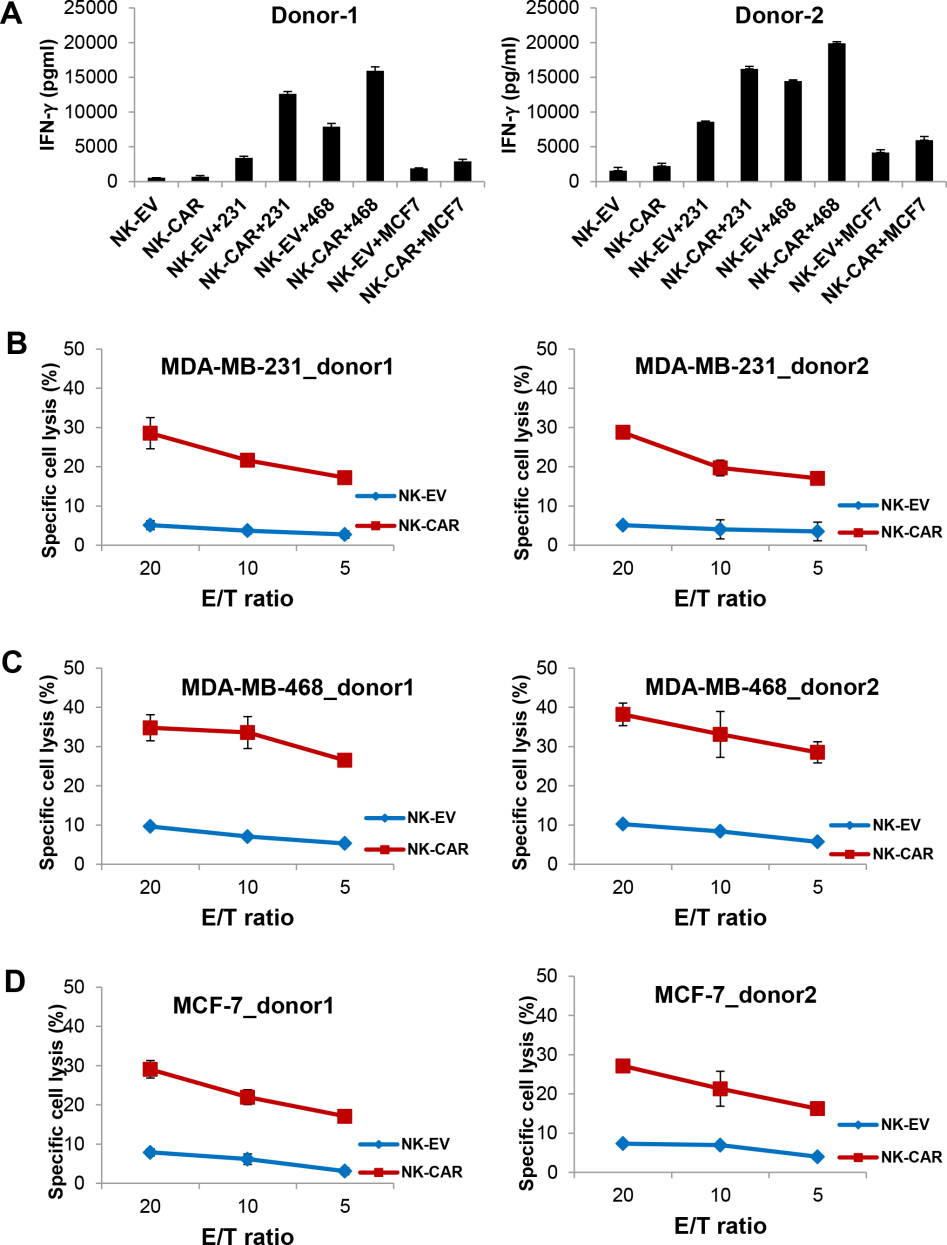
Enhanced cytotoxicity and IFN-γ production of EGFR-CAR primary NK cells when stimulated with EGFR^+^ breast cancer cells (**A**) IFN-γ release by empty vector (EV)-transduced or EGFR-CAR-transduced primary NK cells in the absence or presence of MDA-MB-231, MDA-MB-468 or MCF-7 cells using a standard ELISA assay. (**B**–**D**) Cytotoxic activity of empty vector (EV)-transduced or EGFR-CAR-transduced primary NK cells against MDA-MB-231 (B), MDA-MB-468 (C), or MCF-7 (D) cells using a standard chromium-51 release assay. (E, effect cell; T, target cell).

We generated a second-generation EGFR-CAR construct in the pCDH lentiviral backbone. This construct sequentially contains a signal peptide, EGFR scFv, a hinge region, CD28, and CD3ζ. NK-92 and primary NK cells from healthy donors were transduced with the CAR-expressing lentiviruses and sorted based on expression of GFP by the vector. We performed flow cytometric analysis using a goat anti-mouse F(ab′)_2_ antibody that recognized the scFv portion of anti-EGFR. Figure 2A shows the expression of EGFR-CAR on the surface of EGFR-CAR-transduced NK-92 cells, which was undetectable on NK-92-EV cells (NK-92 cells transduced with the empty vector pCDH). Next, we explored whether EGFR-CAR expression could confer NK-92 and primary NK cells with enhanced IFN-γ production and cytolytic activity. We observed that EGFR-CAR-transduced NK-92 and primary NK cells (Supplementary Figure 1) secreted significantly higher levels of IFN-γ when co-cultured with MDA-MB-231 cells or MDA-MB-468 cells as compared to their corresponding effector cells transduced with an empty vector (Figures 2B, 3A). Interestingly, this change in IFN-γ secretion was less discernible when MCF-7 cells with a lower level of EGFR expression were used as targets. Moreover, upon co-culture with these three cell lines, we observed a significant increase in the cytotoxic activity of EGFR-CAR-transduced NK-92 and primary donor derived NK cells compared to that of mock-transduced NK-92 effector cells (Figure 2C–2E) or primary NK cells, respectively (Figure 3B–3D). Using CD69 surface expression to measure effector cell activation, we also observed that tumor cells with EGFR expression can activate EGFR-CAR-transduced NK-92 cells, with higher activation when MDA-MB-231 and MDA-MB-468 cells were used than when MCF-7 cells were used. We also detected expression of CD27, another NK cell activation marker, and observed that CD27 was not expressed on the surface of EGFR-CAR NK-92 cells (Supplementary Figure 2).

### Lysis of breast cancer cell lines by oHSV-1

**Figure 4:**
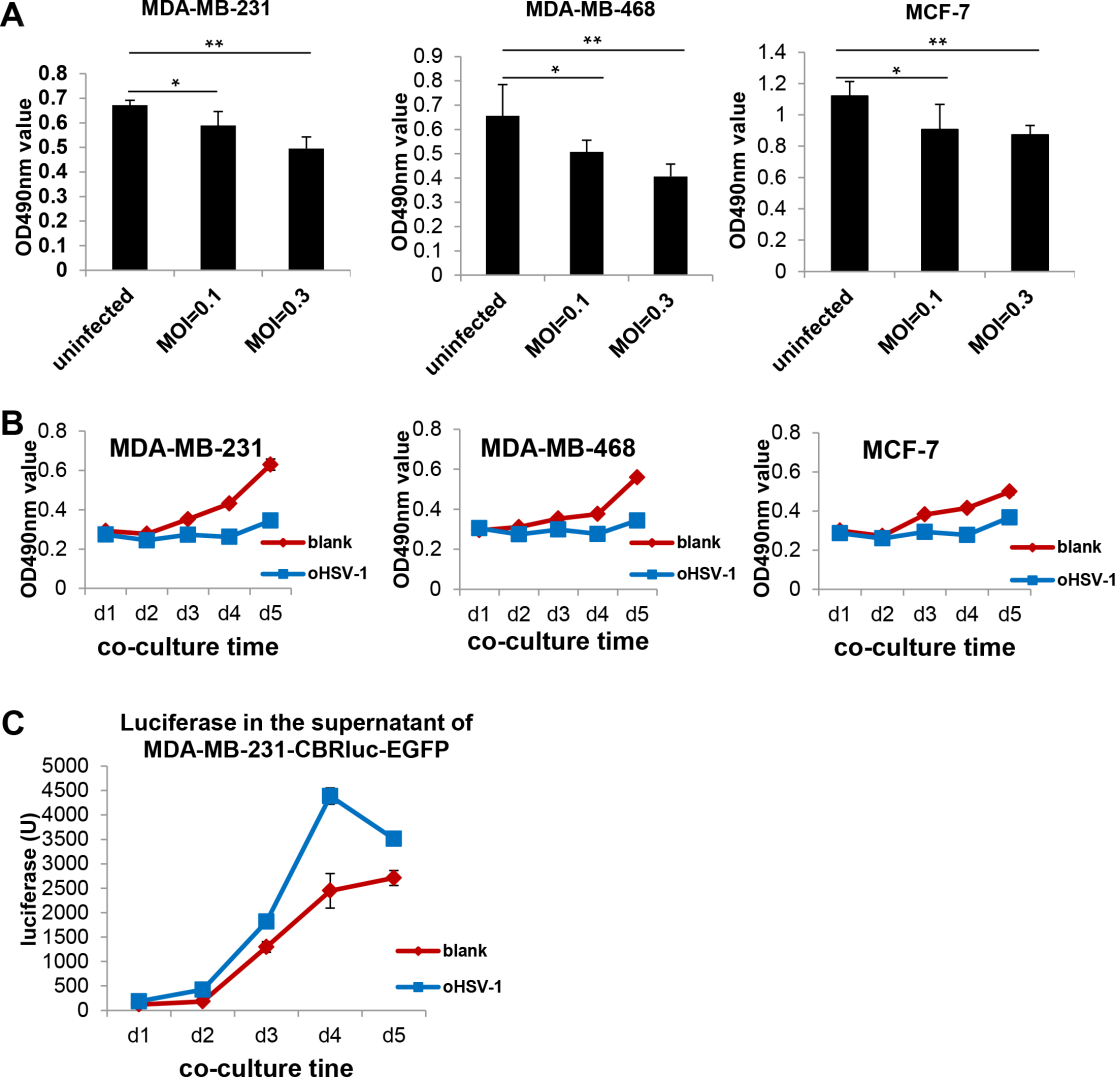
oHSV-1 alone can lyse and eradicate breast cancer cell line tumor cells (**A**) Dose-dependent cytotoxicity of oHSV-1 to breast cancer cell lines (MDA-MB-231, MDA-MB-468 or MCF-7) after co-culture for 48 h and detected by MTS. *P < 0.05; **P < 0.01. (**B**) MTS assays of oHSV-1 cytotoxicity against breast cancer cell lines, MDA-MB-231, MDA-MB-468 or MCF-7, after co-cultured of them for different time periods. (**C**) Measurement of luciferase levels in the media of the co-culture of MDA-MB-231-CBRluc-EGFP cells and oHSV-1.

Previous data from our group and others demonstrated that oHSV-1 can lyse glioblastoma cells but spare normal cells [11, 14, 15]. In the current study, we explored whether oHSV-1 alone could lyse and destroy breast cancer cells, which have the capability of trafficking into the brain to form metastatic brain tumors. As shown in Figure 4A, oHSV-1 reduced the viability of MDA-MB-231, MDA-MB-468, and MCF-7 cells in a dose-dependent fashion after co-culture for 48 h, and this effect was observed at different time points (Figure 4B). Microscopic analysis showed that oHSV-1 alone could lyse these breast cancer cell line cells after co-culture for 4 days (Supplementary Figure 3A). This was confirmed using luciferase-expressing MDA-MB-231 cells (MDA-MB-231-CBRluc-EGFP), in which a higher level of luciferase was detected in the supernatants from the group with oHSV-1 infection compared to the mock-infected group (*P* < 0.01 at day 4) (Figure 4C). Meanwhile, oHSV-1 did not lyse or induce apoptosis of EGFR-CAR NK-92 effector cells, as determined by a microscopic examination (Supplementary Figure 3B).

### EGFR-CAR NK-92 cells in combination with oHSV-1 result in more efficient eradication of cancer cells *in vitro*

**Figure 5:**
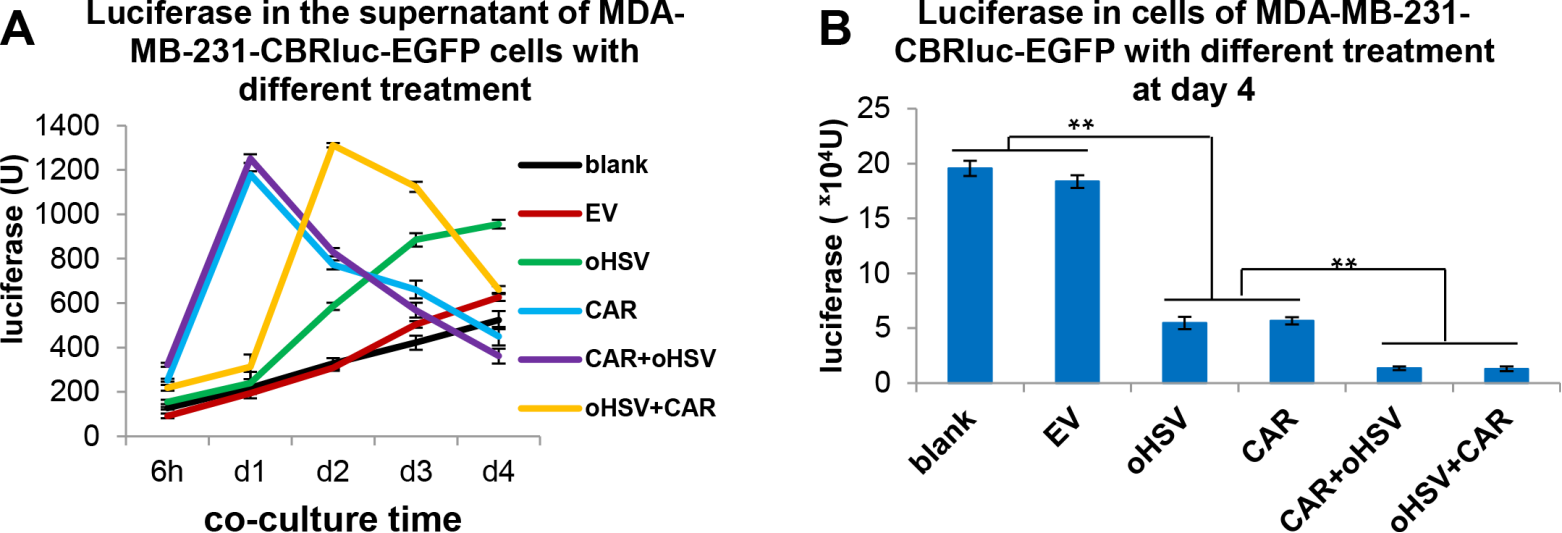
The combinational treatment of EGFR-CAR NK-92 cells and oHSV-1 results in more efficient eradication of breast cancer tumor cells *in vitro* (**A**) Tumor cells were treated with CAR cells alone, oHSV-1 alone, EGFR-CAR NK-92 cells for 4 h followed by oHSV-1 (CAR + oHSV), or oHSV for 4 h followed by EGFR-CAR NK-92 cells (oHSV + CAR). Eradication of MDA-MB-231 tumor cells expressing CBRluc-EGFP was measured by luciferase release to supernatants at different time points. (**B**) Regardless of the order, the EGFR-CAR NK-92 cells in combination with oHSV-1 (CAR + oHSV or oHSV + CAR) eradicated more MDA-MB-231 tumor cells than EGFR-CAR NK-92 cells alone (CAR) or oHSV-1 alone (oHSV), determined by the relative light units of luciferase remained in the MDA-MB-231-CBRluc-EGFP cells on day 4 after co-cultured. **P < 0.01. Data are representative of three independent experiments.

When MDA-MB-231 cells were treated with EGFR-CAR NK-92 cells alone or in combination with oHSV-1 (either treatment with EGFR-CAR NK-92 cells for 4 h followed by oHSV-1 treatment or vice versa), MTS assays indicated that the MDA-MB-231 cell line was efficiently killed under all circumstances; however, the combination of EGFR-CAR NK-92 cells with oHSV-1 resulted in more efficient killing (data not shown). We then assessed killing by measuring luciferase activity in the supernatants of MDA-MB-231-CBRluc-EGFP cells following different treatments. Luciferase was found to be degraded quickly (not shown), and thus, the luciferase assay allowed us to determine dynamic, real-time killing rather than accumulative killing. Based on this, we observed that EGFR-CAR NK-92 cells alone and EGFR-CAR NK-92 cells combined with oHSV-1 caused more rapid lysis than oHSV-1 alone (Figure 5A). When measuring luciferase activity in the remaining MDA-MB-231-CBRluc-EGFP cells (cell pellets) after a co-culture for 4 days, we found that EGFR-CAR NK-92 cells alone, oHSV-1 alone, or EGFR-CAR-NK-92 cells combined with oHSV-1 all led to substantial killing of MDA-MB-231-CBRluc-EGFP cells, and EGFR-CAR NK-92 cells combined with oHSV-1 regardless of the order was more effective than the monotherapies (Figure 5B). Similar results were observed by microscopic examination (Supplementary Figure 4). EGFR-CAR NK-92 cells quickly destroyed some of the MDA-MB-231 cells, but a subset of these cells still maintained their original cell shape and integrity even after 5 days. oHSV-1 first caused the target cancer cells to aggregate, then the cells were gradually lysed (Supplementary Figure 4). However, the combination of EGFR-CAR NK-92 cells and oHSV-1 resulted in more robust cell killing, especially in the CAR NK-92 cells followed by oHSV-1 treatment group (row 5, Supplementary Figure 4). Of note, consistent with ^51^Cr release assays (Figure 2C) and luciferase data (Figure 5B), microscopic analysis demonstrated that MDA-MB-231 cells were resistant to killing by NK-92-EV cells.

### EGFR-CAR NK-92 cells combined with oHSV-1 lead to more efficient killing of MDA-MB-231 tumor cells in an intracranial model

**Figure 6:**
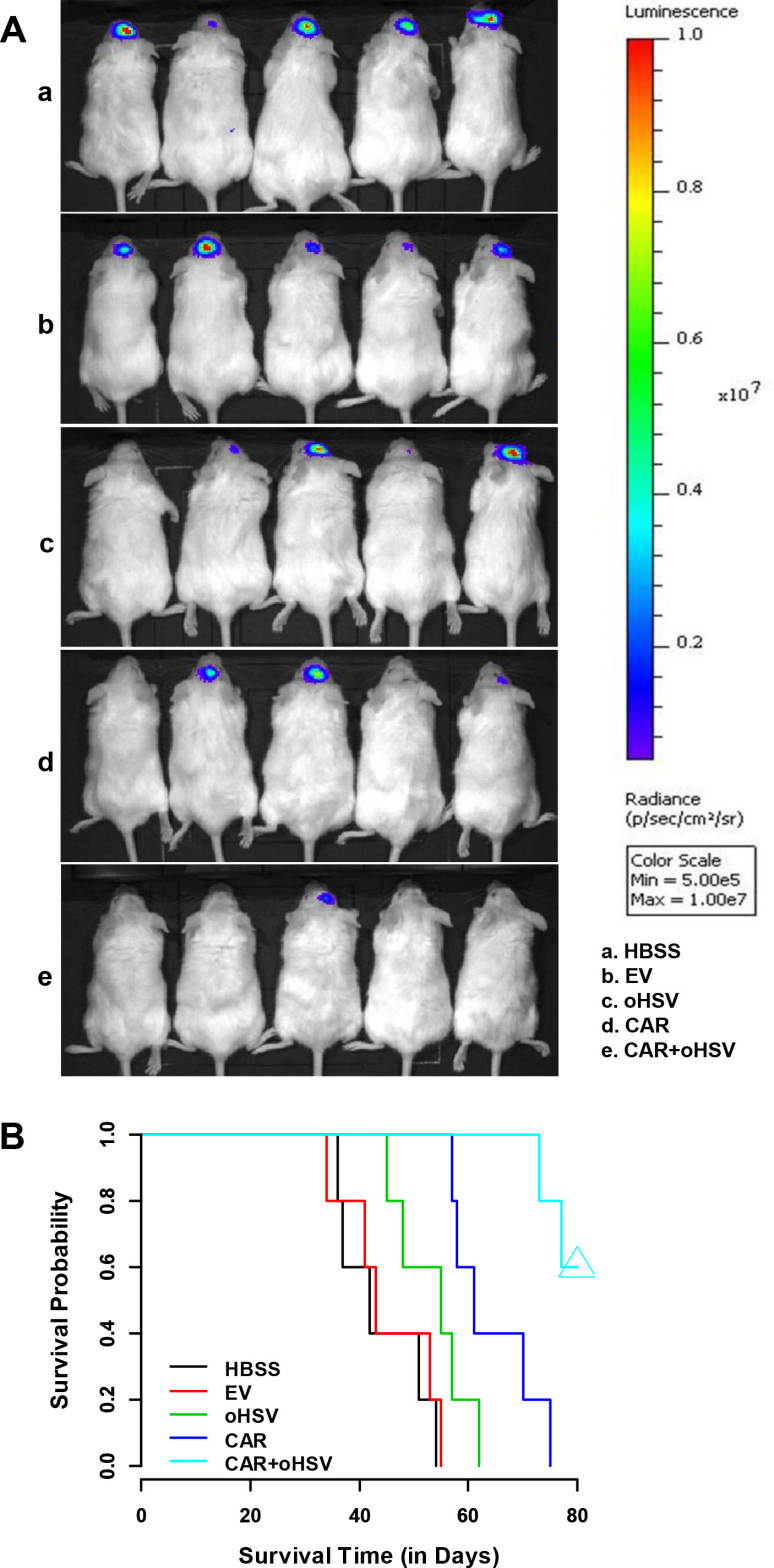
EGFR-CAR transduced NK-92 cells inhibit MDA-MB-231 tumor growth with prolonged survival of the tumor-bearing mice (**A**) Brain bioluminescence imaging of mice bearing BCBM tumors. NSG mice were inoculated with MDA-MB-231-CBRluc-EGFP cells via stereotaxic injection (day 0). 10 days after inoculation, mice were intracranially infused once with EGFR-CAR NK-92, oHSV-1, NK-92-EV, or HBSS. The mice of combined treatment group were injected with oHSV-1 on day 15. Four weeks after inoculation with MDA-MB-231-CBRluc-EGFP cells, the mice were intraperitoneally infused with D-luciferin and imaged using the *in vivo* Imaging System. (**B**) MDA-MB-231-CBRluc-EGFP tumor-bearing mice were intratumorally treated with EGFR-CAR NK-92 cells followed by oHSV-1 injection (CAR + oHSV), EGFR-CAR NK-92 cells alone (CAR), oHSV-1 alone, or HBSS control. As a result, EGFR-CAR NK-92 cells followed by oHSV-1 injection showed significantly increased overall survival than the rest of treatments as determined by Kaplan-Meier survival curves (*n* = 5 for each group).

To further support the potential therapeutic application of EGFR-CAR NK-92 cells, oHSV-1 alone, or the combination of both, we examined their antitumor activity *in vivo*. We established an intracranial model of breast cancer by implanting MDA-MB-231-CBRluc-EGFP cells into the brains of NSG mice. The expression of beetle red luciferase in the cells enabled us to monitor tumor growth via *in vivo* bioluminescence imaging. To minimize potential systemic toxicity, we injected the non-irradiated EGFR-CAR NK-92 cells or oHSV-1 intratumorally at day 10 post-tumor cell implantation and oHSV-1 at day 15 for the group of EGFR-CAR NK-92 combined with oHSV-1. As shown in Figure 6A and Supplementary Figure 5, mice that received either EGFR-CAR NK-92, oHSV-1, or their combination had significantly reduced tumor growth compared to those injected with mock-transduced NK-92-EV or vehicle (HBSS). Importantly, the reduction in tumor growth was more obvious in mice treated with EGFR-CAR NK-92 combined with oHSV-1 than in those treated with EGFR-CAR NK-92 alone or oHSV-1 alone. In agreement with these data, the mice treated with EGFR-CAR NK-92 plus oHSV-1 survived significantly longer than those treated with oHSV-1 alone (*P* < 0.01), mock-transduced NK-92 (*P* < 0.001), or HBSS (*P* < 0.001), while the difference between the group of EGFR-CAR NK-92 plus oHSV-1 and EGFR-CAR NK-92 alone showed the same trend and was at the border of the significance threshold (*P* = 0.0757). The median survival time of the five groups for EGFR-CAR NK-92 combined with oHSV-1, EGFR-CAR NK-92, oHSV-1, NK-92-EV and HBSS were 80, 61, 55, 43, and 42 days, respectively (Figure 6B).

## DISCUSSION

The overall goal of cancer therapy is to achieve durable effects and ultimately find a cure with minimal toxicity. Compared to hematological malignancies, successful treatment of solid cancers faces more barriers, especially for metastatic solid cancers such as BCBMs. The treatment of BCBM patients is extremely challenging and still lacks effective strategies [3]. Although the problems such as tumor lysis syndrome and cytokine release syndrome have been encountered, CAR T cells have been successful in the clinic for treatment of several types of hematological cancers [16] and are now used as treatment for solid cancers including glioblastoma [17, 18]. OVs have been studied since 1991 [19] for solid tumors, and the first oncolytic virotherapy (talimogene laherparepvec, T-VEC) was approved by the FDA in Oct 2015 for the treatment of melanoma. However, both preclinical and clinical studies demonstrate that as a single agent, OV is not very effective. Using several lines of reasoning outlined below, we hypothesized that EGFR-CAR NK cells combined with oHSV-1 would be a more effective therapeutic strategy for BCBMs than either treatment alone. Our data demonstrate that intratumoral administration of EGFR-CAR NK-92 cells, oHSV-1, or the combination of both into mice pre-inoculated with MDA-MB-231 cells led to antitumor efficacy and their combination resulted in more efficient suppression of tumor growth and significantly longer survival of tumor-bearing mice.

We believe this combination will be an effective approach for BCBMs at least in part because of its potential to target CSCs, a cell population responsible for relapse, treatment resistance, and metastasis in most if not all cancers [20]. In fact, in a separate study using glioblastoma as a model, we showed that EGFR-CAR NK cells effectively eradicate glioblastoma (GBM) CSC both *in vitro* and *in vivo* [21]. It is also known that OVs, including oHSV, are capable of infecting and killing CSCs [22]. Gralow et al. reported that not all breast cancer cells within a tumor mass possess the same metastatic potential, and only a small subset of CSCs disseminate to specific sites in the body [23]. Metastasis is a complex process whereby a cell must incorporate tumorigenicity with invasion, extravasation, and migration to secondary sites. Therefore, CSCs must possess each of these abilities to metastasize [24]. Al-Hajj et al. showed that the CSCs of breast cancer displayed a CD44^+^/CD24^−/low^ phenotype, originally defined as a tumor-initiating population, might be more closely associated with metastatic cancer cells [25]. Li et al. demonstrated that oHSVs were highly cytotoxic to the CD44^+^/CD24^−/low^ breast CSCs *in vitro*, and more importantly, they could significantly inhibit the growth of tumors derived from the CD44^+^/CD24^−/low^ population in mice compared with mock treatment [22]. Deng et al. also demonstrated that CAR T cells targeting EpCAM were able to kill prostate CSCs [26]. At least some breast CSCs express EGFR [27–29] and thus can be targeted by EGFR-CAR NK cells.

We also believe our combinational approach would be effective in treating BCBMs because it accommodates heterogeneous tumor populations, as they exist in essentially all types of cancers. That is, EGFR-expressing cells are targeted by EGFR-CAR NK cells, but oHSV-1 also can kill EGFR-negative tumor cells. It is well known that breast cancer is heterogeneous for EGFR, PR, and HER2 expression. Although the breast cancer cell lines used in our experiments express wild-type EGFR, each expresses this to a different degree. Meanwhile, they have different gene expression profiling [MDA-MB-231 and MDA-MB-468: ER–, PR–, HER2– (triple negative); MCF-7: ER+, PR+/−, HER2–] and distinct biological behaviors. Triple-negative breast cancer (TNBC) is associated with an aggressive natural history as well as an increased susceptibility to metastasis [30]. Patients with TNBC lack the “traditional” therapeutic targets and have a poorer prognosis than other types of breast cancer. In fact, median survival for TNBC is only 4.9 months [3]. The combinational approach we described in this study should target BCBMs of both non-TNBC and TNBC, as oHSV is effective for the general BCBM population, while EGFR-CAR NK cells are more effective in targeting the EGFR+ populations.

It is challenging to find a suitable tumor antigen to target in cancer, especially in solid tumors. In our current study, we chose to target EGFR on BCBMs because EGFR plays an important role in tumor cell proliferation, motility, and survival [7, 31]. EGFR has two forms on the surface of cells, wild-type EGFR (wtEGFR) and mutant EGFR (EGFRvIII). However, EGFRvIII expression is rare in breast cancer when compared to wtEGFR. Fan et al. reported that among 58 glioblastoma tumors, 83% (48/58) stained for wtEGFR by IHC and 19% (11/58) were positive for EGFRvIII, and all EGFRvIII-positve tumors also express wtEGFR [32]. With regard to breast cancer, Gojis et al. reported 30 cases of breast cancer patients with metastasis and found positive wtEGFR protein expression in 12 patients (40%). There was no statistically significant difference of EGFR expression between primary cancer and brain metastasis [6]. Since the EGFR-CAR NK-92 cells that we generated were able to target both wtEGFR and EGFRvIII, they will be more broadly applicable than agents targeting EGFRvIII alone, particularly in the setting of BCBMs. Using intracranial administration as demonstrated here, EGFR-CAR NK cells are expected to be relatively safe, as EGFR expression is almost undetectable in human brain tissues [33]. In future studies, it will be important to determine the threshold of EGFR expression required to initiate the killing by EGFR-CAR NK cells, as others have found that expression levels of wtEGFR can vary over a three log range in breast cancer patient samples and cell lines [34].

Much of cellular immunotherapy so far has focused on T cells. We focused on CAR NK cells, as they have not yet been used in the clinics for cancer treatment but have shown preclinical potential [35, 36]. CAR NK cells also may have a lower risk of producing tumor lysis syndrome and cytokine release syndrome as seen with CAR T cells in the clinic. Moreover, the use of CAR NK cells may avoid graft-versus-host disease (GVHD), and thus could be used in the allogeneic setting. Importantly, arming the NK-92 cell line with CARs recognizing antigens on multiple different tumor cells provides us with an opportunity to generate renewable, off-the-shelf products that could be more affordable and accessible to a broad population of cancer patients.

Both primary brain cancer such as glioblastoma and metastatic brain cancer such as BCBMs are devastating diseases without effective therapies. Due to the nature of their location, approaches are also limited. We undertook the OV approach, since it represents an exciting biological approach to cancer therapy with a distinct mechanism of action when compared with conventional cancer therapeutics in that OVs selectively replicate and ultimately lyse tumor cells [8, 37]. Kambara et al. reported that the oHSV-1 mutant (rQNestin34.5), which we used in this study, was engineered by expressing ICP34.5 under the control of a synthetic nestin promoter, so it can selectively replicate in nestin positive tumors [38]. Meanwhile, Sihto et al. reported that primary tumors with brain as the first metastatic site more frequently express nestin than those with the first metastasis at other sites (15.8% vs. 3.7%) [39]. Thus, oHSV-1, EGFR-CAR NK cells, and their combination are capable of selectively targeting and destroying tumor cells of BCBMs and all of them, especially the combination, may have an optimal efficacy in patients with tumor cells expressing EGFR.

We investigated the combination of oHSV with CAR NK cells because, as described, oHSV as a single agent is not very effective for treatment of cancer. We previously found that one limiting factor is the NK cell response to oHSV at early infection stages [11, 14]. Preclinical studies by others have demonstrated enhanced efficacy when oHSV is combined with cytotoxic anticancer drugs [40]. Recent research has uncovered promising combinatorial approaches employing oHSV as well as other agents that are mechanism-based and often exhibit synergistic anti-cancer effects [41]. Our study showed that EGFR-CAR NK-92 cells can quickly target and attack breast cancer cells while oHSV-1 can slowly but constantly infect and destroy the cancer cells. EGFR-CAR NK-92 cells can usually recognize and attack target cells in several hours, but they can survive only several days because they have to be irradiated as the cell line was originally established from a patient with non-Hodgkin's lymphoma. An irradiation dose of 1000 cGy has been optimized to suppress proliferation of NK-92 cells while maintaining full cytotoxic activity up to 48 hours post irradiation [42]. On the contrary, it may take about 4 days for oHSV to enter into target cells, replicate, and destroy the tumor cells, even though its effects can last for a long time. In addition, CAR-modified NK cells may destroy the tumor tissue structure and decrease the connection between tumor cells, increase the permeability of cancer cell membranes, and therefore enhance virus distribution and replication in cancer cells when combined with oHSV-1. Finally, our previous study showed that NK cells can eradicate oHSV at early infection stages. For all of these reasons, we propose to administer EGFR-CAR NK cells first, followed by infusion of oHSV in several days. The early infusion of EGFR-CAR NK-92 cells can quickly control tumor growth and decrease tumor size in the brain, providing potential rapid relief of neurologic symptoms and intracranial hypertension [43]. oHSV administered at a later time is able to kill the remaining cancer cells and continuously induce the patient's immune response against cancer cells. This strategy can prevent oHSV from being eliminated by irradiated EGFR-CAR NK-92, since the irradiated CAR cells may lose their killing capacity at the time when oHSV is adminstered. Using this strategy, we intend to control the development of tumor lesions and minimize the probability of tumor relapse, eventually prolonging survival and improving quality of life in patients with metastatic brain cancer such as BCBMs or with primary brain cancer such as glioblastoma.

In conclusion, we developed a novel and promising strategy by using intracranial injection of EGFR-CAR-modified human NK cells followed by oHSV-1 administration to target human EGFR positive brain cancers such as BCBMs. Our current study provides an experimental basis for the future clinical application of this strategy.

## MATERIALS AND METHODS

### Cell culture

Human breast cancer cell lines MDA-MB-231, MDA-MB-468, and MCF-7, as well as 293T and Phoenix cells, were cultured in DMEM (Invitrogen, Grand Island, NY) and supplemented with 10% FBS, penicillin (100 U/ml), and streptomycin (100 μg/ml) (all from Invitrogen). Human NK cell line NK-92 and primary NK cells (obtained from the American Red Cross in Columbus) were maintained in RPMI-1640 (Invitrogen) supplemented with 20% FBS, penicillin (100 U/ml), streptomycin (100 μg/ml), and 200 IU/mL recombinant human (rh) IL-2 (Gold Biotechnology, MO).

### Mice

Six to eight-week-old NOD.Cg-*Prkdc^scid^ Il2rg^tm1Wjl^*/SzJ (NSG) mice were obtained from Jackson Laboratories (Bar Harbor, ME). All animal work was approved by The Ohio State University Animal Care and Use Committee. Mice were monitored daily for disease progression and sacrificed when they became moribund with neurologic impairments or showed obvious weight loss.

### Generation of EGFR-CAR lentiviral construct

The anti-EGFR single chain variable fragment (scFv) was derived from DNA sequences encoding a specific monoclonal antibody against both wtEGFR and EGFRvIII [44]. The VH-linker-VL fragment was incorporated in frame with the CD28-CD3ζ portion incised from a retroviral vector. The entire anti-EGFR-scFv-CD28-CD3ζ fragment was then ligated into a lentiviral vector designated as pCDH-CMV-MCS-EF1-copGFP (System Biosciences, Mountain View, CA) to generate the pCDH-EGFR-scFv-CD28-CD3ζ (pCDH-EGFR-CAR) construct.

### Lentiviral production and transduction of NK-92 cells

To produce lentivirus for infection of NK-92 cells, 293 T cells were co-transfected with the aforementioned pCDH-EGFR-scFv-CD28-CD3ζ plasmid or a mock pCDH vector together with packaging constructs pCMV-VSVG and pCMV-DR9 using calcium phosphate transfection reagent (Promega, Madison, WI). The transfection and infection procedures were modified from a previously published protocol [35].

### Generation of MDA-MB-231 cells stably expressing CBRluc-EGFP

MDA-MB-231 cells stably expressing CBRluc-EGFP was generated by retroviral transfection with the ΔU3CBRluc-EGFP vector (a generous gift from Dr. JF DiPersio) following a previously published protocol [35]. EGFP positive breast cancer cells were then sorted using a FACS Aria II cell sorter (BD Biosciences, San Jose, CA) and expanded, yielding MDA-MB-231-CBRluc-EGFP cells.

### Flow cytometry analysis

To determine EGFR expression on the surface of breast cancer cell lines, cells were incubated with the mouse monoclonal anti-human EGFR (clone H11, DAKO) antibody, followed by staining with APC-conjugated goat anti-mouse IgG secondary antibody. The surface expression of EGFR-CAR was assessed by flow cytometry as described previously [35].

### Hematoxylin and eosin (HE) staining and immunohistochemistry (IHC) assay

Paraffin-embedded sections of tumor tissues from patients with both primary breast cancer and brain metastasis were stained with HE or with anti-wild-type-EGFR antibody (1:2000, DAK-H1-WT; Agilent Technologies, Santa Clara, CA) for IHC. An automatic immunostainer (BenchMark XT, Ventana Medical Systems, Tucson, AZ) was used according to the manufacturer's instructions. Sections were visualized and photographed by a Leica laser confocal microscope (SP5Wetzlar, Germany).

### Cytotoxicity assay

A standard 4-h ^51^Cr release assay was performed as described previously [45]. The percentage of specific cell lysis was calculated using the standard formula: 100 × (cpm experimental release – cpm spontaneous release) / (cpm maximal release – cpm spontaneous release).

### IFN-γ release assay

1 × 10^6^ target cells were incubated with equal numbers of effector cells in the wells of 96-well V-bottom plates for 24 h. Cell-free supernatants were assayed for IFN-γ secretion by enzyme-linked immunosorbent assay (ELISA) using a kit from R & D Systems (Minneapolis, MN) according to the manufacturer's protocol. Data depicted in figures represent mean values of triplicate wells from one of three representative experiments with similar results.

### MTS assay

Breast cancer cell line cells (5 × 10^3^) were seeded in 96 well flat bottom culture plates and incubated at 37°C in DMEM medium containing 10% FBS. At the end of treatment, cell viability was determined using a rapid, tetrazoliumbased MTS (3-(4,5-dimethylthiazol-2-yl)-5-(3-carboxymethoxyphenyl)-2-(4-sulfophenyl)-2H-tetrazolium, inner salt) colorimetric assay (CellTiter 96 cell proliferation assay kit; Promega, Madison, WI) according to the manufacturer's instructions [46]. All experiments were performed at least in triplicates on three separate occasions.

### Luciferase assay

MDA-MB-231-CBRluc-EGFP cells (5 × 10^3^) were seeded in 96well flat bottom culture plates and incubated at 37°C in DMEM medium containing 10% FBS with different treatments. At different time points, 20 μL of the culture media were collected directly for luciferase assays using the Dual-Glo Luciferase Assay System (Promega), as described previously [47]. At day 4, cell pellets were rinsed twice with PBS, and then lysed with 30 μL of 1× passive lysis buffer (Promega). Lysates were pelleted by centrifugation (13,000 rpm, 1 minute) and the supernatant was collected to measure luciferase activity.

### Treatment of breast cancer brain invasion in NSG mice

NSG mice were anesthetized and fixed in a stereotactic apparatus, and 1 × 10^5^ MDA-MB-231-CBRluc-EGFP cells in 2 μL Hank's buffered salt solution (HBSS) were injected into mouse brain on day 0, where a burr hole was drilled 2 mm laterally and 1 mm anteriorly to the right bregma to a depth of 3.25 mm. On day 10, the mice were injected intratumorally with 2 × 10^6^ effector cells, i.e. EGFR-CAR-transduced NK-92 cells (NK-92-EGFR-CAR) or empty vector-transduced NK-92 cells (NK-92-EV) in 5 μL HBSS. The oHSV-1 alone group was injected intratumorally with 2 × 10^5^ plaque-forming units (pfu) oHSV-1 (rQNestin34.5) [36] in 5 μL HBSS. Mice treated with 5 μl HBSS were used as a control. On day 15, mice in the CAR plus oHSV-1 treatment group were intratumorally injected with 2 × 10^5^ pfu oHSV-1. Mice were monitored daily and euthanized when they showed signs of morbidity. Four weeks after inoculation with MDA-MB-231-CBRluc-EGFP cells, the mice were intraperitoneally (i.p.) infused with D-luciferin (150 mg/kg body weight; Gold Biotechnology, St. Louis, MO, USA), anesthetized with isoflurane, and imaged using the *in vivo* Imaging System (IVIS-100, PerkinElmer, Waltham Massachusetts, USA) with living image software (PerkinElmer).

### Statistics

The unpaired Student's *t* test was used to compare two independent groups for continuous endpoints if normally distributed with or without data transformation. One-way ANOVA was used to compare among three or more groups. For survival data, Kaplan-Meier analysis was used to estimate survival functions and log-rank test was used to compare the survival between two groups. All tests were two-sided. *P* values were adjusted for multiple comparisons using Holm's procedure. A *P* value of less than 0.05 was considered statistically significant.

## SUPPLEMENTARY MATERIALS FIGURES



## References

[1] DeSantis CE, Lin CC, Mariotto AB, Siegel RL, Stein KD, Kramer JL, Alteri R, Robbins AS, Jemal A (2014). Cancer treatment and survivorship statistics, 2014. CA Cancer J Clin.

[2] Boogerd W (1996). Central nervous system metastasis in breast cancer. Radiother Oncol.

[3] Niikura N, Hayashi N, Masuda N, Takashima S, Nakamura R, Watanabe K, Kanbayashi C, Ishida M, Hozumi Y, Tsuneizumi M, Kondo N, Naito Y, Honda Y (2014). Treatment outcomes and prognostic factors for patients with brain metastases from breast cancer of each subtype: a multicenter retrospective analysis. Breast Cancer Res Treat.

[4] Barrett DM, Singh N, Porter DL, Grupp SA, June CH (2014). Chimeric antigen receptor therapy for cancer. Annu Rev Med.

[5] Uherek C, Tonn T, Uherek B, Becker S, Schnierle B, Klingemann HG, Wels W (2002). Retargeting of natural killer-cell cytolytic activity to ErbB2-expressing cancer cells results in efficient and selective tumor cell destruction. Blood.

[6] Gojis O, Kubecova M, Rosina J, Vranova J, Celko M, Frajerova D, Zmrhal J, Zahumensky J, Bacova T, Baca V, Mandys V, Kucera E (2013). Expression of selected proteins in breast cancer brain metastases. Folia Histochem Cytobiol.

[7] Brand TM, Iida M, Luthar N, Starr MM, Huppert EJ, Wheeler DL (2013). Nuclear EGFR as a molecular target in cancer. Radiother Oncol.

[8] Russell SJ, Peng KW, Bell JC (2012). Oncolytic virotherapy. Nat Biotechnol.

[9] Shen Y, Nemunaitis J (2006). Herpes simplex virus 1 (HSV-1) for cancer treatment. Cancer Gene Ther.

[10] Robert HI, Andtbacka FAC, Amatruda Thomas, Senzer Neil N, Chesney Jason, Delman Keith A (2013). OPTiM: A randomized phase III trial of talimogene laherparepvec (T-VEC) versus subcutaneous (SC) granulocyte-macrophage colony-stimulating factor (GM-CSF) for the treatment (tx) of unresected stage IIIB/C and IV melanoma. J Clin Oncol.

[11] Alvarez-Breckenridge CA, Yu J, Price R, Wojton J, Pradarelli J, Mao H, Wei M, Wang Y, He S, Hardcastle J, Fernandez SA, Kaur B, Lawler SE (2012). NK cells impede glioblastoma virotherapy through NKp30 and NKp46 natural cytotoxicity receptors. Nat Med.

[12] Han J, Chen X, Chu J, Xu B, Meisen WH, Chen L, Zhang L, Zhang J, He X, Wang QE, Chiocca EA, Kaur B, Caligiuri MA (2015). TGFbeta Treatment Enhances Glioblastoma Virotherapy by Inhibiting the Innate Immune Response. Cancer Res.

[13] Maude SL, Frey N, Shaw PA, Aplenc R, Barrett DM, Bunin NJ, Chew A, Gonzalez VE, Zheng Z, Lacey SF, Mahnke YD, Melenhorst JJ, Rheingold SR (2014). Chimeric antigen receptor T cells for sustained remissions in leukemia. N Engl J Med.

[14] Alvarez-Breckenridge CA, Yu J, Price R, Wei M, Wang Y, Nowicki MO, Ha YP, Bergin S, Hwang C, Fernandez SA, Kaur B, Caligiuri MA, Chiocca EA (2012). The histone deacetylase inhibitor valproic acid lessens NK cell action against oncolytic virus-infected glioblastoma cells by inhibition of STAT5/T-BET signaling and generation of gamma interferon. J Virol.

[15] Meisen WH, Wohleb ES, Jaime-Ramirez AC, Bolyard C, Yoo JY, Russell L, Hardcastle J, Dubin S, Muili K, Yu J, Caligiuri M, Godbout J, Kaur B (2015). The Impact of Macrophage- and Microglia-Secreted TNFalpha on Oncolytic HSV-1 Therapy in the Glioblastoma Tumor Microenvironment. Clin Cancer Res.

[16] Lee DW, Kochenderfer JN, Stetler-Stevenson M, Cui YK, Delbrook C, Feldman SA, Fry TJ, Orentas R, Sabatino M, Shah NN, Steinberg SM, Stroncek D, Tschernia N (2015). T cells expressing CD19 chimeric antigen receptors for acute lymphoblastic leukaemia in children and young adults: a phase 1 dose-escalation trial. Lancet.

[17] Guest RD, Kirillova N, Mowbray S, Gornall H, Rothwell DG, Cheadle EJ, Austin E, Smith K, Watt SM, Kuhlcke K, Westwood N, Thistlethwaite F, Hawkins RE (2014). Definition and application of good manufacturing process-compliant production of CEA-specific chimeric antigen receptor expressing T-cells for phase I/II clinical trial. Cancer Immunol Immunother.

[18] Katz SC, Burga RA, McCormack E, Wang LJ, Mooring W, Point GR, Khare PD, Thorn M, Ma Q, Stainken BF, Assanah EO, Davies R, Espat NJ (2015). Phase I Hepatic Immunotherapy for Metastases Study of Intra-Arterial Chimeric Antigen Receptor-Modified T-cell Therapy for CEA+ Liver Metastases. Clin Cancer Res.

[19] Martuza RL, Malick A, Markert JM, Ruffner KL, Coen DM (1991). Experimental therapy of human glioma by means of a genetically engineered virus mutant. Science.

[20] Reya T, Morrison SJ, Clarke MF, Weissman IL (2001). Stem cells, cancer, and cancer stem cells. Nature.

[21] Han J, Chu J, Keung Chan W, Zhang J, Wang Y, Cohen JB, Victor A, Meisen WH, Kim SH, Grandi P, Wang QE, He X, Nakano I (2015). CAR-Engineered NK Cells Targeting Wild-Type EGFR and EGFRvIII Enhance Killing of Glioblastoma and Patient-Derived Glioblastoma Stem Cells. Sci Rep.

[22] Li J, Zeng W, Huang Y, Zhang Q, Hu P, Rabkin SD, Liu R (2012). Treatment of breast cancer stem cells with oncolytic herpes simplex virus. Cancer Gene Ther.

[23] Gralow JR (2005). Optimizing the treatment of metastatic breast cancer. Breast Cancer Res Treat.

[24] Lawson JC, Blatch GL, Edkins AL (2009). Cancer stem cells in breast cancer and metastasis. Breast Cancer Res Treat.

[25] Al-Hajj M, Wicha MS, Benito-Hernandez A, Morrison SJ, Clarke MF (2003). Prospective identification of tumorigenic breast cancer cells. Proc Natl Acad Sci U S A.

[26] Deng Z, Wu Y, Ma W, Zhang S, Zhang YQ (2015). Adoptive T-cell therapy of prostate cancer targeting the cancer stem cell antigen EpCAM. BMC Immunol.

[27] Lee CH, Wu YT, Hsieh HC, Yu Y, Yu AL, Chang WW (2014). Epidermal growth factor/heat shock protein 27 pathway regulates vasculogenic mimicry activity of breast cancer stem/progenitor cells. Biochimie.

[28] Hardt O, Wild S, Oerlecke I, Hofmann K, Luo S, Wiencek Y, Kantelhardt E, Vess C, Smith GP, Schroth GP, Bosio A, Dittmer J (2012). Highly sensitive profiling of CD44+/CD24-breast cancer stem cells by combining global mRNA amplification and next generation sequencing: evidence for a hyperactive PI3K pathway. Cancer Lett.

[29] Foley J, Nickerson NK, Nam S, Allen KT, Gilmore JL, Nephew KP, Riese DJ (2010). EGFR signaling in breast cancer: bad to the bone. Semin Cell Dev Biol.

[30] Herold CI, Anders CK (2013). New targets for triple-negative breast cancer. Oncology (Williston Park).

[31] Hoadley KA, Weigman VJ, Fan C, Sawyer LR, He X, Troester MA, Sartor CI, Rieger-House T, Bernard PS, Carey LA, Perou CM (2007). EGFR associated expression profiles vary with breast tumor subtype. BMC Genomics.

[32] Fan QW, Cheng CK, Gustafson WC, Charron E, Zipper P, Wong RA, Chen J, Lau J, Knobbe-Thomsen C, Weller M, Jura N, Reifenberger G, Shokat KM (2013). EGFR phosphorylates tumor-derived EGFRvIII driving STAT3/5 and progression in glioblastoma. Cancer Cell.

[33] Maenpaa A, Kovanen PE, Paetau A, Jaaskelainen J, Timonen T (1997). Lymphocyte adhesion molecule ligands and extracellular matrix proteins in gliomas and normal brain: expression of VCAM-1 in gliomas. Acta Neuropathol.

[34] Rae JM, Scheys JO, Clark KM, Chadwick RB, Kiefer MC, Lippman ME (2004). EGFR and EGFRvIII expression in primary breast cancer and cell lines. Breast Cancer Res Treat.

[35] Chu J, He S, Deng Y, Zhang J, Peng Y, Hughes T, Yi L, Kwon CH, Wang QE, Devine SM, He X, Bai XF, Hofmeister CC (2014). Genetic modification of T cells redirected toward CS1 enhances eradication of myeloma cells. Clin Cancer Res.

[36] Castriconi R, Daga A, Dondero A, Zona G, Poliani PL, Melotti A, Griffero F, Marubbi D, Spaziante R, Bellora F, Moretta L, Moretta A, Corte G (2009). NK cells recognize and kill human glioblastoma cells with stem cell-like properties. J Immunol.

[37] Gupta SK, Gandham RK, Sahoo AP, Tiwari AK (2015). Viral genes as oncolytic agents for cancer therapy. Cell Mol Life Sci.

[38] Kambara H, Okano H, Chiocca EA, Saeki Y (2005). An oncolytic HSV-1 mutant expressing ICP34. 5 under control of a nestin promoter increases survival of animals even when symptomatic from a brain tumor. Cancer Res.

[39] Sihto H, Lundin J, Lundin M, Lehtimaki T, Ristimaki A, Holli K, Sailas L, Kataja V, Turpeenniemi-Hujanen T, Isola J, Heikkila P, Joensuu H (2011). Breast cancer biological subtypes and protein expression predict for the preferential distant metastasis sites: a nationwide cohort study. Breast Cancer Res.

[40] Meisen WH, Dubin S, Sizemore ST, Mathsyaraja H, Thies K, Lehman NL, Boyer P, Jaime-Ramirez AC, Elder JB, Powell K, Chakravarti A, Ostrowski MC, Kaur B (2015). Changes in BAI1 and nestin expression are prognostic indicators for survival and metastases in breast cancer and provide opportunities for dual targeted therapies. Mol Cancer Ther.

[41] Kanai R, Rabkin SD (2013). Combinatorial strategies for oncolytic herpes simplex virus therapy of brain tumors. CNS Oncol.

[42] Schonfeld K, Sahm C, Zhang C, Naundorf S, Brendel C, Odendahl M, Nowakowska P, Bonig H, Kohl U, Kloess S, Kohler S, Holtgreve-Grez H, Jauch A (2015). Selective inhibition of tumor growth by clonal NK cells expressing an ErbB2/HER2-specific chimeric antigen receptor. Mol Ther.

[43] Soffietti R, Ruda R, Mutani R (2002). Management of brain metastases. J Neurol.

[44] Hayashi H, Asano R, Tsumoto K, Katayose Y, Suzuki M, Unno M, Kodama H, Takemura S, Yoshida H, Makabe K, Imai K, Matsuno S, Kumagai I (2004). A highly effective and stable bispecific diabody for cancer immunotherapy: cure of xenografted tumors by bispecific diabody and T-LAK cells. Cancer Immunol Immunother.

[45] Yu J, Mao HC, Wei M, Hughes T, Zhang J, Park IK, Liu S, McClory S, Marcucci G, Trotta R, Caligiuri MA (2010). CD94 surface density identifies a functional intermediary between the CD56bright and CD56dim human NK-cell subsets. Blood.

[46] Hayon T, Dvilansky A, Shpilberg O, Nathan I (2003). Appraisal of the MTT-based assay as a useful tool for predicting drug chemosensitivity in leukemia. Leuk Lymphoma.

[47] Fu X, Tao L, Rivera A, Williamson S, Song XT, Ahmed N, Zhang X (2010). A simple and sensitive method for measuring tumor-specific T cell cytotoxicity. PLoS One.

