# Supplementary material for: A combinational therapy of EGFR-CAR NK cells and oncolytic herpes simplex virus 1 for breast cancer brain metastases
Source: Oncotarget. 2016 Apr 1;7(19):27764–77. doi: 10.18632/oncotarget.8526 (PMC5053686; doi:10.18632/oncotarget.8526)
Supplement: Supplementary file 1 [file oncotarget-07-27764-s001.pdf]

A combinational therapy of EGFR-CAR NK cells and oncolytic herpes simplex virus 1 for breast cancer brain metastases

Supplementary Materials

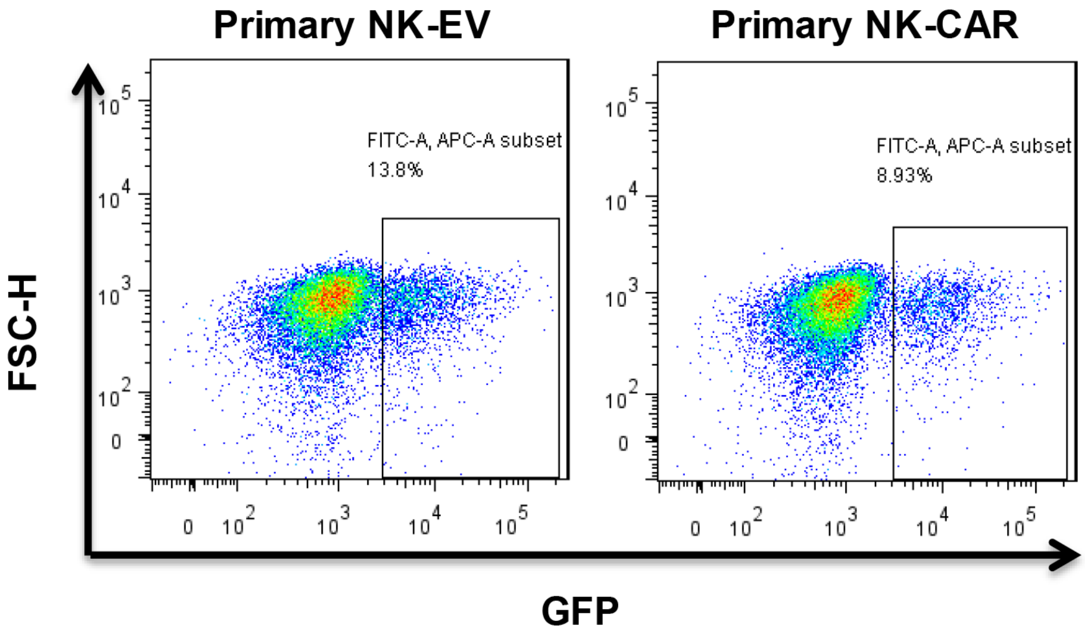

Supplementary Figure S1: The transduction efficiency of lentiviruses in human primary NK cells. The percentage of GFP (+) cells was determined by flow cytometry after human primary NK cells were infected with EGFR-CAR lentiviruses.

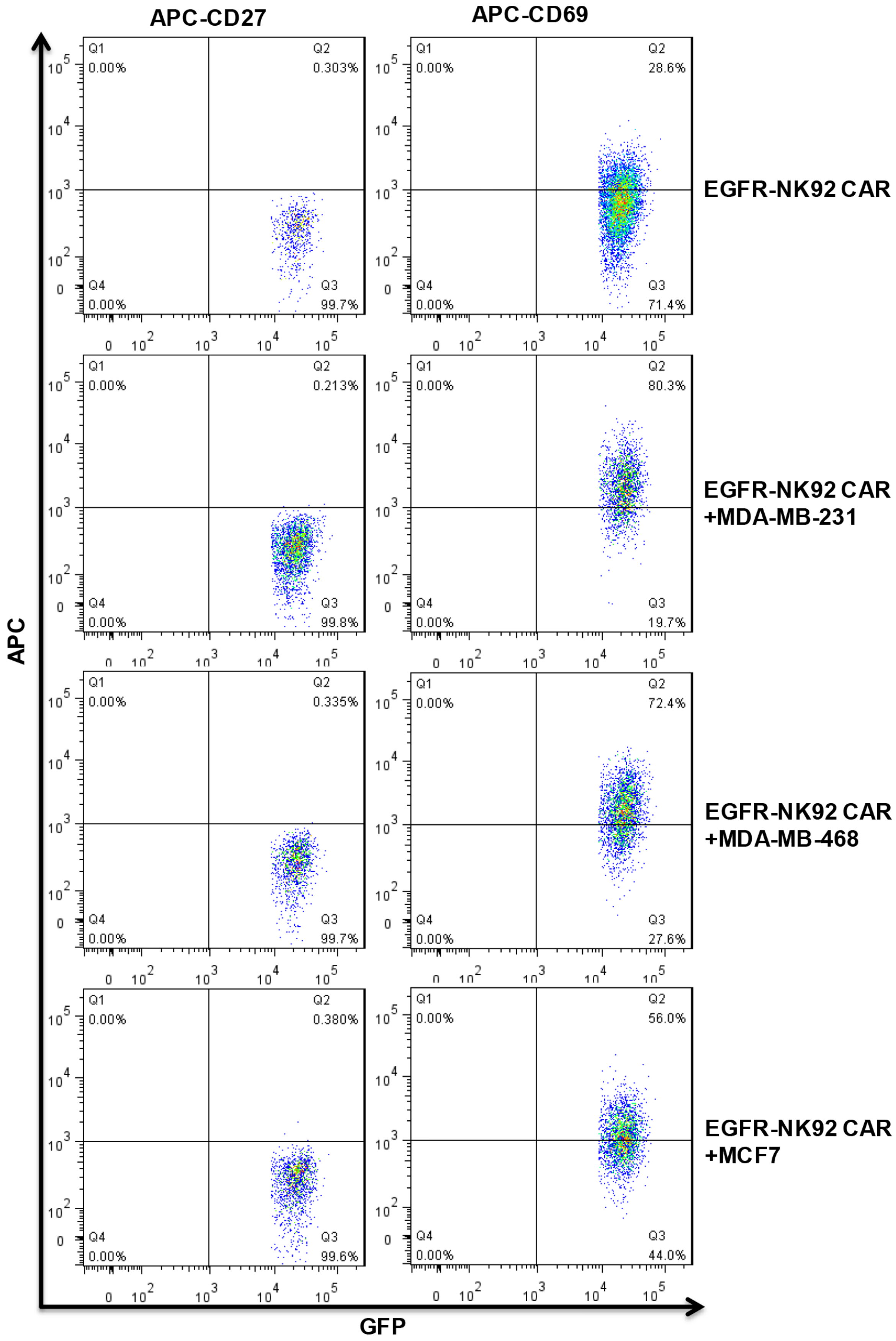

Supplementary Figure S2: The change in expression of activation markers on the surface of EGFR-CAR NK-92 cells in response to breast cancer cells. Surface expression of CD27 and CD69 in EGFR-CAR NK-92 cells was determined after co-culture with breast cancer cells (MDA-MB-231, MDA-MB-468 and MCF-7) overnight by flow cytometry.

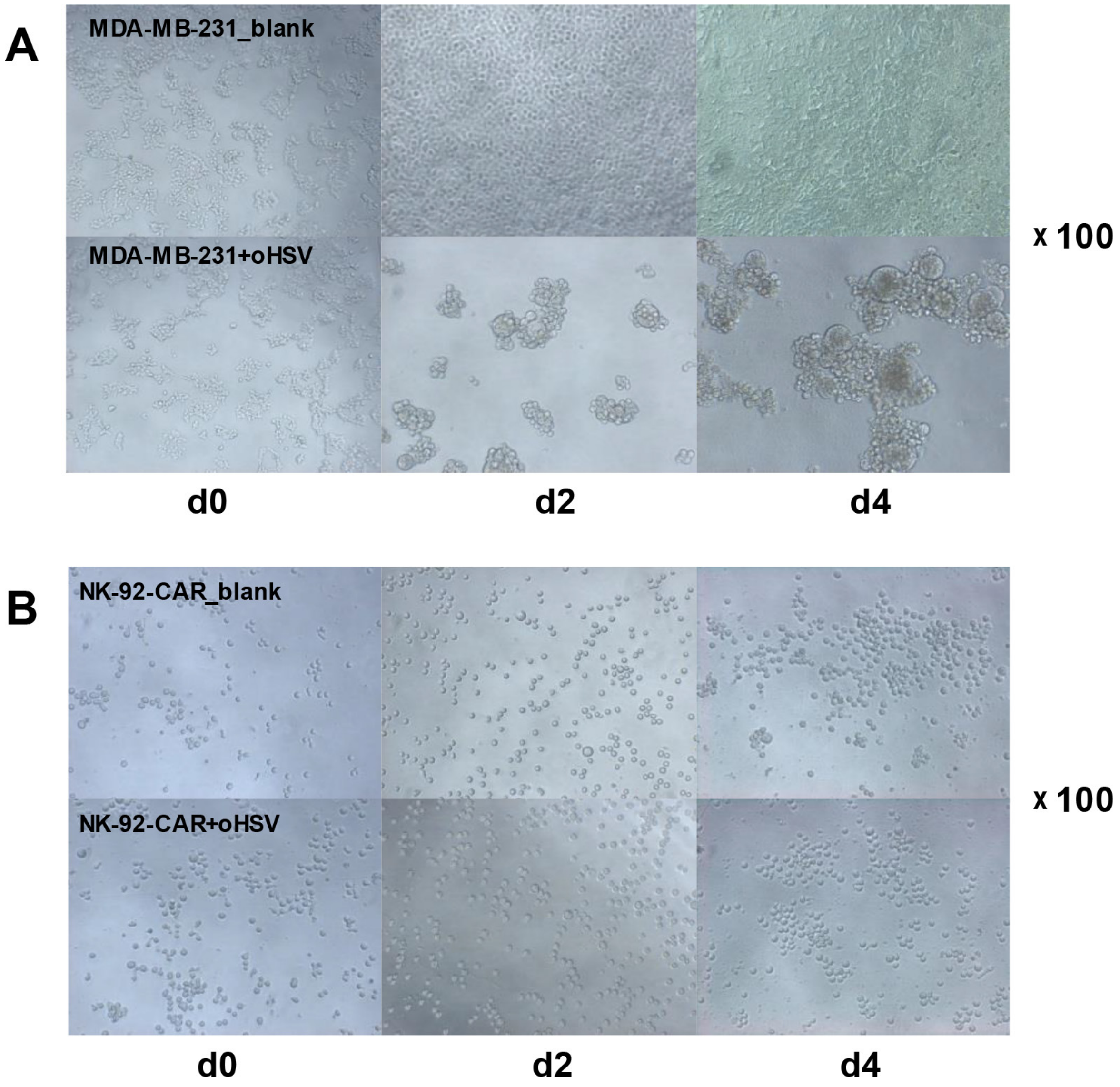

Supplementary Figure S3: Lysis of breast cancer cells by oHSV alone. (**A**) Lysis of breast cancer cell line (MDA-MB-231) by oHSV after co-culture for 4 days, demonstrated by the bright images under microscope. (**B**) EGFR-CAR NK-92 cells were treated with oHSV-1 for 4 days, and microscopic examination showed that oHSV-1 had no obvious effect on the proliferation and viability of EGFR-CAR NK-92 cells.

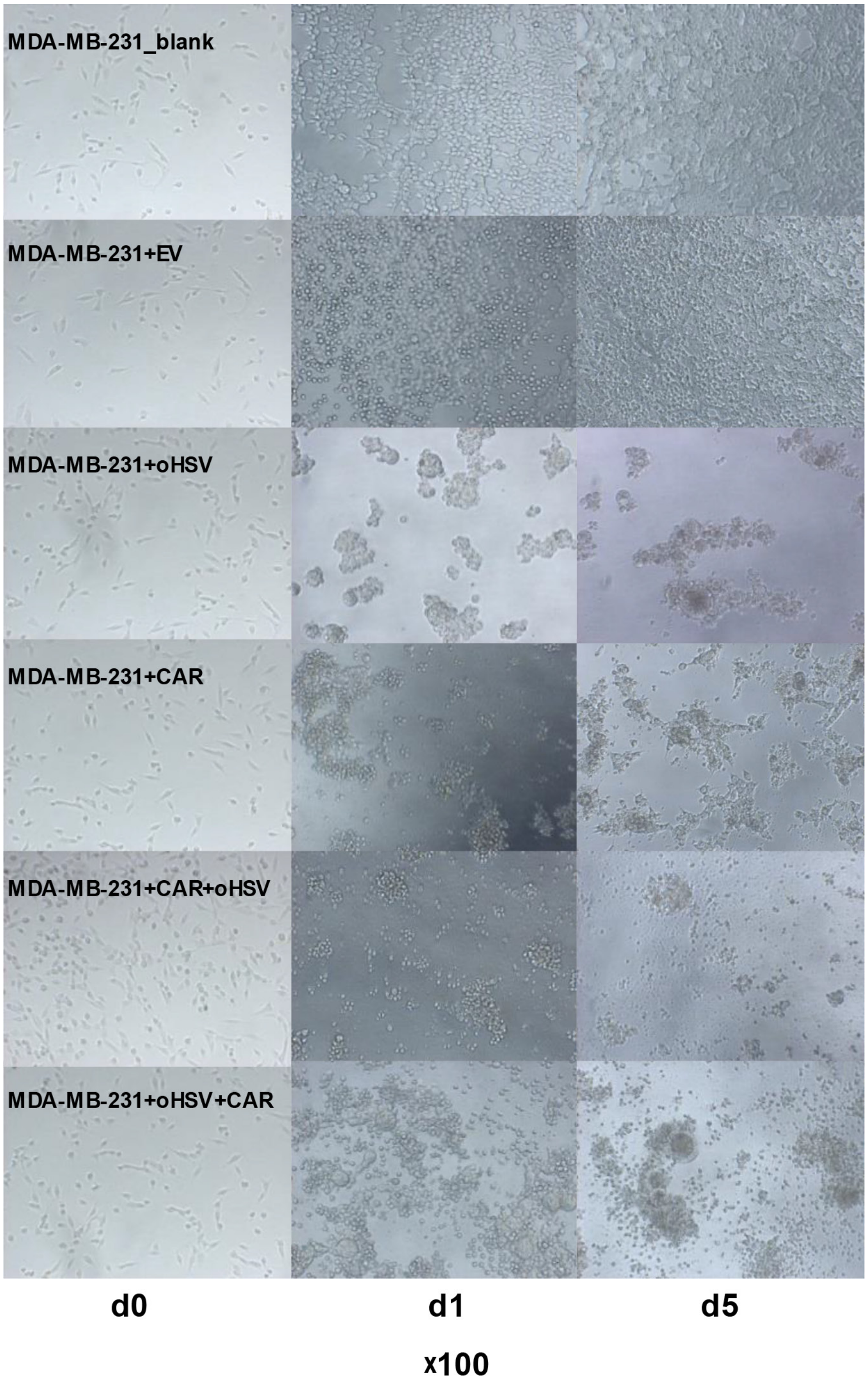

Supplementary Figure S4: Lysis of breast cancer cell line (MDA-MB-231) by oHSV, EGFR-CAR NK-92 cells, and their combination. “MDA-MB-231+CAR+oHSV” denotes treatment of EGFR-CAR NK-92 cells for 4 h, followed by oHSV-1 treatment. “MDA-MB-231+oHSV+CAR” denotes treatment of oHSV-1 for 4 h, followed by treatment of EGFR-CAR NK-92 cells.

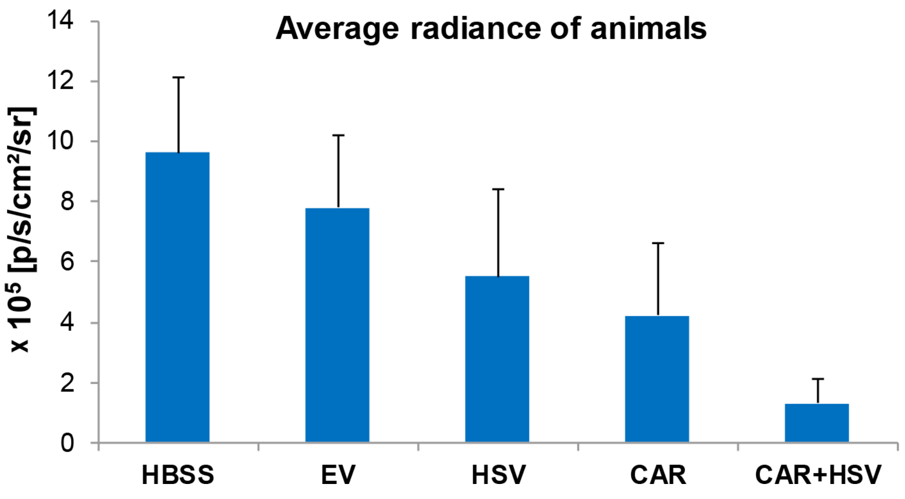

Supplementary Figure S5: Quantification of emitted photons from intracranial xenograft GBM tumors treated with vehicle, NK-92 cells, oHSV, EGFR-CAR NK-92 cells, and the combination of oHSV and EGFR-CAR NK-92 cells.
